# Behavioral Economics in People Management: A Critical and Integrative Review

**DOI:** 10.3390/bs16010065

**Published:** 2026-01-01

**Authors:** Antonio M. Espín, Jesús M. García-Martínez

**Affiliations:** 1 Department of Applied Economics, University of Granada, Campus de Cartuja S/N, 18071 Granada, Spain; 2 Department of Social Psychology and Quantitative Psychology, University of Barcelona, 08007 Barcelona, Spain; jesusmaria.garcia@ub.edu

**Keywords:** people management, behavioral incentives, behavior change, organizational psychology, economic games, human resources, nudges, preferences

## Abstract

In recent years, behavioral economics has revolutionized various fields, including finance, marketing, and public policy. Its application in people management, however, remains an emerging area of exploration. By integrating psychological insights into economic decision-making, behavioral economics offers a nuanced understanding of human behavior, essential for designing effective HR practices. While many of the concepts are not new in organizational behavior research and related fields, thanks to the incorporation of formalized models of choice, behavioral economics brings analytical clarity to domains traditionally studied through descriptive or qualitative methods in the behavioral sciences. This review article delves into how behavioral economics can shed light on key aspects of people management, focusing on five domains: incentives, decision-making, leadership, personalization, and organizational change. We offer a critical overview integrating some of the most well-known findings with applicability in these areas as well as promising avenues for future research. One of the main conclusions is that behavioral economics offers a powerful lens to approach people management, but also that behavioral principles need to be understood in depth (beyond average effects, for example) as generalization is often flawed, claiming for personalized solutions and interventions grounded on comprehensive perspectives.

## 1. Introduction

Behavioral economics is a strand of economics that introduces psychological aspects, broadly speaking, into economic analysis. At its core, behavioral economics departs from the traditional assumption of full rationality and instead embraces the psychological and social dimensions that shape individual and collective choices, often by focusing on behaviors that deviate from the prescriptions of rational choice theory (Simon, 1955; Ellsberg, 1961). This paradigm shift in economics has revealed that even small contextual features—such as framing, defaults, or perceived fairness—can have substantial effects on behavior (Thaler & Sunstein, 2008). In recent years, behavioral economics has become a powerful lens through which scholars and practitioners understand decision-making in diverse domains, such as finance, consumer behavior and public policy (Thaler & Sunstein, 2008; Kahneman, 2011; Loewenstein & Chater, 2017).

Yet despite its profound impact across disciplines, the integration of behavioral economics into people management remains relatively underexplored. Given that “people management”—a catch-all term encompassing human resources, talent management, and other organizational decision-making processes involving employees—addresses interactions among individuals within organizations where economic exchanges occur, the behavioral economics perspective offers a natural framework for its theoretical and empirical advancement. This article aims to offer a critical review of how insights from behavioral economics can enrich and update people management practices. Rather than filling an absolute void, our objective is to synthesize and operationalize behavioral economics concepts in ways that complement existing organizational behavior and human resource management approaches.

Note that this is not a systematic or scoping review, and therefore our literature selection follows the authors’ convenience and personal preferences to build our arguments. We present a narrative critical review aimed at integrating existing findings rather than showing new empirical results. Our contribution lies in synthesizing behavioral economics mechanisms and translating them into actionable, organization-focused insights for people management. Our intention is thus not to replace existing theories in organizational behavior, but to complement them by importing formal behavioral-economic constructs that allow deeper modeling of heterogeneity and contextual effects. Whenever possible, we redirect the reader to other sources of knowledge, or previous studies or reviews focused on particular elements. More specifically, the limited prior work explicitly integrating behavioral economics into people management necessarily leads us to draw upon a small set of foundational contributions in this intersection, including some of our own previous work. In addition, we provide a glossary of concepts in Appendix A to help readers from diverse backgrounds follow our arguments without compromising the flow of the article.

**Figure 1:**
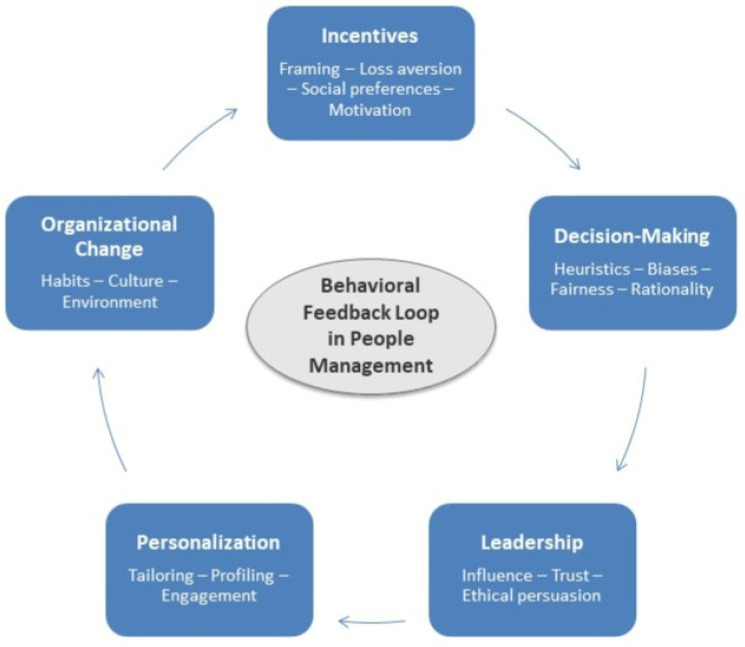
Integrative framework: Behavioral economics in people management.

While academic research in organizational behavior and related fields has acknowledged the importance of social and emotional factors, many real-world practices continue to rely on simplified, one-size-fits-all assumptions. These “outdated behavioral models” include, for example, uniform pay-for-performance schemes that overlook individual motivational differences (Hackman & Oldham, 1976) or leadership training programs designed without accounting for measurable variations in risk tolerance, fairness preferences, or intrinsic motivation (Ryan & Deci, 2000). Behavioral economics offers tools to refine these practices by incorporating stylized facts, empirically derived parameters, and individualized behavioral profiles, giving rise to what can be labelled as “organizational behavioral economics” (Camerer & Malmendier, 2007; Houdek & Koblovský, 2017). Figure 1 shows the framework used here, focusing on the five dimensions considered in the next sections, namely, incentives, decision-making, leadership, personalization, and organizational change. The framework displayed in Figure 1 should not be seen as a static taxonomy but as a behavioral feedback model that illustrates how these areas interact in a reinforcing manner within organizations.

For instance, in incentive design, framing a bonus as a potential loss rather than a gain—aligned with measured levels of loss aversion—can significantly increase motivation among certain employee segments. In leadership development, matching leaders’ influence strategies to followers’ reciprocity norms and fairness concerns can improve team cohesion and performance. These applications, which will be discussed in detail in the next sections, illustrate how behavioral profiling and evidence-based tailoring can transform existing HR practices into more precise and effective interventions. A recurring construct throughout this review is indeed behavioral profiling, which refers to the use of experimentally validated behavioral measures to capture stable individual differences relevant for organizational decision-making.

Behavioral economics, as a branch of economics and the behavioral sciences, departs from the traditional assumption of full rationality and integrates psychological and social dimensions into economic models of decision-making. These dimensions—such as loss aversion, time preferences, and reciprocity—are quantified through experimental and empirical methods, allowing them to be directly applied to the design and evaluation of people management practices. In this sense, behavioral economics complements rather than replaces broader behavioral science perspectives.

In fact, behavioral economics bridges the gap between traditional economic models—grounded in formal mathematical structures and utility theory—and the rich, context-sensitive insights from the behavioral sciences. It thus provides tools to model and predict behavior while accounting for the emotional, social, and cognitive dimensions often emphasized in organizational behavior research. Behavioral economics contributes to the behavioral sciences by introducing formal analytical tools—such as utility functions, decision models under uncertainty, and game-theoretic reasoning—that help structure and quantify behavioral hypotheses. This formalization enhances theoretical precision in domains like organizational behavior, which have traditionally relied more on qualitative or descriptive approaches. Conversely, behavioral economics benefits from the behavioral sciences by integrating constructs such as emotions, social identity, and environmental cues; dimensions that were historically underappreciated in economics but are central to understanding behavior in organizational settings. This bidirectional integration allows behavioral economics to contribute novel perspectives to people management, offering not only predictive and prescriptive models of behavior, but also a framework to design empirically grounded interventions that reflect the real-world complexity of human motivation and decision-making.

Section 2 is devoted to “Incentives in the Workplace”. Incentives are fundamental tools in people management, designed to motivate and align employee behavior with organizational goals. Section 2 explores different types of incentives, including monetary rewards/penalties (both absolute and relative) and non-monetary benefits (social perks, recognition programs, and flexible schedules). Behavioral economics reveals how individuals perceive and react to incentives, often diverging from traditional economic assumptions of rationality. Moreover, Section 2 examines the potential pitfalls of incentive systems and proposes strategies to design fair and effective reward structures that consider each individual’s preferences and motivational forces.

Section 3 is about “Processes and Decision-Making in People Management”. People management involves a series of critical decisions, from recruitment and performance evaluation to promotions and terminations. Behavioral biases, such as implicit discrimination and the halo effect, often undermine objectivity in these processes. Section 3 explores how behavioral economics can mitigate such biases through structured decision-making frameworks and nudges. For example, anonymized CVs can reduce bias in recruitment, while behavior-based performance metrics minimize subjective judgments in appraisals. Additionally, it discusses the ethical dimensions of workplace policies, emphasizing the importance of balancing efficiency with fairness in decisions such as wage revisions and layoffs.

Later on, we introduce “Leadership and Behavioral Influence”. Effective leadership is a cornerstone of organizational success. Behavioral economics offers insights into how leaders can influence their teams through principles such as social proof, authority, and reciprocity. Behavioral leadership integrates insights from behavioral economics and psychology, recognizing leaders as fallible decision-makers shaped by context. Effective leaders act as behavioral architects, designing systems and incentives that align with real human behavior—not idealized norms. Section 4 examines various leadership styles, comparing transformational and transactional approaches, and their impact on employee behavior. It also highlights strategies for managing interpersonal dynamics, including the judicious use of punishment and rewards to encourage desired behaviors. By integrating behavioral insights, leaders can foster stronger, more collaborative teams while avoiding common pitfalls like over-reliance on coercive tactics.

Section 5 underscores the importance of “Personalization and Employee Experience”. Understanding individual preferences and motivators is crucial for tailoring HR practices to meet diverse employee needs. Although personalization features prominently throughout the article, Section 5 specifically discusses how behavioral profiling can identify what drives employee engagement and performance, ultimately leading to organizational and employee well-being gains. Personalization not only enhances employee satisfaction but also aligns individual goals with organizational objectives, creating a mutually beneficial dynamic. Employee experience encompasses the entire journey of an individual within an organization, from onboarding to offboarding. Along these lines, behavioral economics provides a framework for designing environments and policies that foster well-being, engagement, and productivity based on personalization. Moreover, linking personalization efforts to broader employee experience strategies ensures a cohesive approach to people management.

Next, our focus is on “Organizational Change Initiatives: A Behavioral Perspective”. Driving behavior change within organizations is often challenging but essential for achieving strategic goals. Change initiatives fail because they rely too heavily on awareness-raising and training while ignoring the behavioral realities of how employees actually operate. Traditional approaches often overlook critical factors like ingrained habits, cognitive biases, and environmental triggers that drive workplace behaviors. Research shows most work routines are automatic, meaning successful change requires deliberately disrupting old patterns and systematically building new ones. To close the persistent intention-behavior gap, organizations must reduce cognitive friction, leverage social and structural supports, and intentionally design for habit formation—moving beyond mere communication to behaviorally informed implementation. Section 6 introduces models such as the COM-B model (Capability, Opportunity, Motivation—Behavior) as a practical framework for understanding and influencing behavior. Behavioral economics principles, such as nudges and habit formation, are discussed in the context of workplace interventions.

In Section 7, we conclude and summarize key future directions by emphasizing that behavioral economics offers a powerful lens through which to view and improve people management practices. Thanks to understanding the psychological drivers of human behavior, organizations can design more effective incentives, make fairer decisions, enhance leadership strategies, and personalize the employee experience. The current review underscores the importance of integrating behavioral insights into HR practices while maintaining ethical considerations. Looking ahead, behavioral economics holds immense potential to shape the future of work, fostering environments where both employees and organizations thrive. This article aims to provide a comprehensive overview of the application of behavioral economics in people management, equipping HR professionals, leaders, and researchers with actionable insights to drive meaningful change.

## 2. Incentives in the Workplace

Designing effective incentive systems remains one of the most persistent challenges in people management. While traditional economic approaches have emphasized monetary compensation as the primary lever for motivating employees, behavioral economics has uncovered the profound influence of psychological, social, and contextual factors in shaping responses to incentives. These insights have shifted attention from the mere amount of the reward (or penalty) to its architecture: how it is framed, delivered, and interpreted (Gneezy et al., 2011; Espín & de Rus Gutiérrez, 2025).

Behavioral economics focuses on understanding the role played by certain psychological factors that tend to push decision making away from traditional rationality. While the term is somewhat slippery, these deviations are often labeled under the umbrella of “cognitive biases”. For example, a central finding in behavioral research is that people do not respond symmetrically to gains and losses. As formalized by prospect theory (Kahneman & Tversky, 1979), most individuals exhibit loss aversion, valuing potential losses more heavily than equivalent gains. This principle has direct implications for incentive design. Bonuses framed as potential losses (e.g., up-front allocations that are clawed back upon underperformance) tend to be more motivating than bonuses framed as gains, as shown in field experiments across various sectors (e.g., Hossain & List, 2012; Fryer et al., 2022). Yet, a loss framing is more likely to affect those individuals displaying stronger loss aversion tendencies and this needs to be considered in the design of incentives since these individual tendencies can be measured (Espín et al., 2017).

Equally important is the role of social comparisons in shaping effort and performance. When incentives are linked to relative performance—such as ranking-based bonuses—they activate comparison-based motivations, which can either fuel healthy competition or induce counterproductive behaviors like sabotage or disengagement among lower performers (Barankay, 2012; Gill et al., 2019). The effects of such systems depend heavily on employees’ social preferences, including inequity aversion (Fehr & Schmidt, 1999) and reciprocity (Falk & Fischbacher, 2006), suggesting that identical incentive structures can yield diverging outcomes across individuals or teams. For example, Bandiera et al. (2005) found that tournament-like schemes can even reduce the performance of (prosocial) workers because they aim to avoid the negative externalities one’s own performance imposes on peers. Thus, as in the case of loss aversion, understanding the motivational forces and social preferences of the individuals stands as a powerful route to designing effective incentive schemes.

A growing body of literature has emphasized the importance of non-monetary and behaviorally informed incentives. Recognition, symbolic rewards, and public visibility of contributions can outperform financial incentives under certain conditions (Bradler et al., 2016; Kube et al., 2012). These findings challenge the assumption that monetary compensation is always the dominant motivator and suggest that intrinsic motivators—autonomy, purpose, relatedness—should be incorporated into reward systems (Deci et al., 1999; Gneezy & Rustichini, 2000).

However, potential counterproductive effects of incentives need to be considered. Poorly designed incentives can undermine motivation through “crowding out” effects, whereby extrinsic rewards displace intrinsic motivation (Frey & Jegen, 2001; Bowles & Polania-Reyes, 2012). This is particularly concerning in prosocial or creative tasks, where instrumentalizing behavior through monetary compensation may lead individuals to reassess the meaning or social value of their work (Ariely et al., 2009). Likewise, the “ratchet effect” in performance-based pay (Lazear, 2000) illustrates how workers may strategically underperform to avoid setting unrealistic future expectations.

Recent scholarship, synthesized in Espín and Martín (2024), highlights the promise of personalized incentives based on measurable behavioral traits (see also Espín & de Rus Gutiérrez, 2025). For example, time-discounting tendencies can inform the optimal frequency of bonus payments, while measures of prosociality or competitiveness may guide decisions between individual or group-based incentives. In a study with software developers, for example, Espín et al. (2019) found that team performance was significantly predicted by the average patience and cooperativeness of team members—suggesting that behavioral assessments can be used to tailor incentive schemes to team composition.

This perspective aligns with a broader shift toward behavioral diagnostics in HR, in which incentive structures are not only based on organizational goals but also on individual-level traits, such as those measured via incentivized experimental tasks. It also resonates with the notion of *hypernudging* (Yeung, 2019): dynamic and personalized interventions enabled by digital tools, AI and behavioral data that allow for real-time calibration of incentives based on observed preferences and behaviors.

In sum, behavioral economics calls for a move beyond “pay-for-performance” logics toward a more nuanced, context-sensitive, and personalized approach to incentive design. Rather than assuming universal responses to monetary compensation, managers should account for heterogeneity in behavioral dispositions, framing effects, and social dynamics to design incentive systems that are both fair and effective.

## 3. Processes and Decision-Making in People Management

People management encompasses a wide array of decisions—the most important being related to recruitment, evaluation, promotion, and dismissal—that are rarely made under conditions of full information or perfect objectivity, which paves the way for a behavioral approach. Behavioral economics has indeed brought to light the extent to which such “people architecture” decisions (Espín & Martín, 2024) are shaped by social preferences, heuristics, implicit biases, and the bounded rationality of decision-makers. It is important to point out that these factors are not necessarily linked to poor decision-making, as there are reasons why departures from strict rationality serve evolutionary functions and can improve our choices through, for example, reduced cognitive load (Gigerenzer, 2004).

One of the most documented issues in recruitment and promotion is implicit discrimination. Randomized field experiments have shown that candidates with identical qualifications receive significantly fewer callbacks when their resumes include names associated with ethnic minorities or women (Bertrand & Mullainathan, 2004; Riach & Rich, 2006). These biases are often unconscious, making them resistant to standard training programs but responsive to choice architecture interventions—such as blind recruitment, structured evaluations, or rule-based candidate shortlisting (Bohnet et al., 2016; Fernández-Mateo & Fernández, 2016). Techniques such as the use of behavioral economic games to assess the traits of candidates while avoiding personal information is a powerful way to prevent recruiters’ biases and gain objectivity in selection/promotion processes, and are in fact being increasingly applied in organizations (Espín, 2025).

Beyond recruitment and promotions, performance evaluations are also susceptible to several kinds of biases, all of which can distort judgments and perpetuate inequality. Structured decision frameworks—such as behavior-based rating scales based on objective tests, objective performance indicators, or peer assessments—can help mitigate the potential negative influence of those psychological factors (Anseel et al., 2009; Campbell & Wiernik, 2015). Behavioral economists have also explored the ethical dimensions of managerial decisions, emphasizing that perceptions of fairness often matter more than absolute outcomes in determining effort and satisfaction (Fehr et al., 2009; Cohn et al., 2015). For example, a salary adjustment perceived as unfair in comparison to peers can erode morale and reduce effort, even if the absolute level of compensation is high, yet this perception is more relevant for individuals displaying social preferences such as inequality aversion.

Building on the latter point, Espín et al. (2017) advocate for incorporating behavioral profiling into managerial processes, arguing that factors like employees’ fairness preferences, tolerance for uncertainty, and social motivations should inform both the design of procedures and the framing of decisions. As in the domain of incentives, behavioral profiling provides a structural mechanism to tailor decision criteria to individual heterogeneity. For instance, layoffs or demotions that are procedurally transparent and accompanied by empathetic communication are less likely to generate disengagement or reputational damage—even when the outcomes are unfavorable. But these effects are often heterogeneous. More specifically, behavioral profiling allows for personalization of processes and incentives, which stands as the most promising avenue to maximize the effectiveness of people management as it does in other areas associated with public and private organizational policies (Mills, 2022; Sunstein, 2024).

Moreover, decision-making in people management must contend with self-selection dynamics. Certain jobs attract individuals with distinct behavioral traits (e.g., risk tolerance or reciprocity, as measured using economic games), which can both benefit and constrain organizational diversity and performance (Friebel et al., 2019; Fouarge et al., 2014). Behavioral economics offers tools to anticipate and manage these dynamics. For instance, the wording of job postings can deter or encourage certain candidates (e.g., women) depending on how traits such as competitiveness or assertiveness are signaled (Espín & de Rus Gutiérrez, 2025; Shurchkov & Eckel, 2018).

Digital tools now enable more sophisticated and personalized interventions. Behavioral dashboards can monitor decision patterns, flag potential biases, and suggest corrective nudges. Yet, as Yeung (2019) and Mills (2022) caution, these tools also pose ethical risks: personalization without transparency can cross into manipulation. Individualized nudging allows designers to manipulate users (and employees in this case), leading them to take actions that go against their interests, and obscure processes amplify this power. Hence, any application of behavioral insights in decision-making must be guided by principles of autonomy, accountability, and procedural fairness.

Ultimately, the promise of behavioral economics in people management lies not in replacing human judgment, but in scaffolding it: creating systems that anticipate our cognitive limitations, promote fairness and adapt to the diverse ways in which people perceive and respond to decisions.

## 4. Leadership and Behavioral Influence

The convergence of behavioral economics, behavioral sciences and leadership theory has enriched our understanding of how real-world leadership works. No longer can leaders be seen as infallible rational actors because they are human, fallible and deeply influenced by their context.

Effective leadership requires the ability to design environments that reflect how people actually behave, not how we wish they would behave. Leaders must act not only as decision-makers but as behavioral architects who create systems, norms, and incentives that support better choices.

Behavioral insights offer powerful tools for designing more ethical, effective and adaptive leadership systems. From “nudging” employee choices to rethinking executive strategy, the practical applications are numerous. As complexity and volatility increase, the need for behaviorally informed leadership becomes more urgent. Organizations that invest in this integrated approach, grounded in empirical data, real human psychology, and behavioral design, will be better positioned to navigate future challenges.

By integrating psychological, social and cognitive factors into the analysis of decision-making, behavioral sciences posit that both leaders and employees are subject to cognitive biases, emotions, social norms and contextual incentives that shape their decisions. This perspective has led to a reconceptualization of leadership, recognizing that effectiveness depends not just on individual traits but also on the capacity to manage complex behavioral environments (Ajzen, 1991; Houdek & Koblovský, 2017).

A scientifically grounded synthesis of behavioral economics applied to leadership is offered by Sutter (2023), emphasizing how cognitive biases and social preferences shape workplace behavior. The author challenges traditional rational-agent leadership models, advocating for context-sensitive interventions, developing key insights including fairness, reciprocity, and bounded rationality as drivers of employee engagement and performance. This work bridges experimental/behavioral economics and leadership practice with clear managerial implications.

### 4.1. Reframing Leadership Theories with Behavioral Insights

Leadership thinking has shifted from focusing on innate traits to emphasizing observable, learnable behaviors as central drivers of organizational success. Today, leadership effectiveness depends less on abstract qualities or formal authority and more on concrete actions that enable organizational transformation. This transition began with the foundational Ohio State and Michigan studies (Kahn & Katz, 1952; Stogdill & Coons, 1957), which showed that leadership effectiveness stems from specific behaviors—particularly those related to tasks and relationships—rather than inherent traits (Yukl, 1989; Brown & Treviño, 2006). Situational Leadership Theory (Hersey & Blanchard, 1969) reinforced this perspective, emphasizing that effective leaders adapt their style to the readiness of their followers. Behavioral economics further supports this view, showing that context, biases, and framing shape decision-making, including within organizations. In this sense, leaders act as “choice architects,” designing environments that optimize decisions and engagement.

The emergence of Transformational Leadership (Burns, 1978; Bass, 1985) expanded the behavioral focus by demonstrating how leaders can inspire, motivate, and elevate followers, fostering cultural change and intrinsic motivation (Bass & Riggio, 2006). More recent models—such as servant leadership—integrate ethical conduct, empowerment, and emotional intelligence, further enriching the behavioral repertoire (Brown & Treviño, 2006). Leader behavior has now become a critical determinant of intervention success. Over the past 15 years, empirical evidence has shown that behavioral orientations such as inspiring, empowering, and adapting strongly influence employee engagement, innovation, and change outcomes (Peng et al., 2020). The progression from adaptive, situational, or transactional leadership (Liden et al., 2025) toward proactive, transformational leadership underscores this trajectory: transformational behaviors build trust, vision, and readiness for change (Bass & Riggio, 2006), leading to higher innovation rates and greater project success, especially in uncertain environments (Jun & Lee, 2023). Nonetheless, adaptive behavioral flexibility remains essential; leaders who adjust directive and supportive behaviors according to context achieve superior results (Liden et al., 2025). CEOs and project leaders who combine inspirational behaviors with situational sensitivity foster resilience and agility, enhancing the effectiveness of complex interventions (Braun et al., 2013).

### 4.2. The Leader as Choice Architect: Shaping Culture and Behavior Through Behavioral Design

The concept of the leader as a choice architect underscores the pivotal role that leadership plays in shaping organizational culture and behavior through behavioral design. The integration of behavioral design into leadership practice offers a framework for shaping culture and behavior within organizations. By acting as choice architects, leaders can harness behavioral insights to foster environments that promote better decisions, stronger engagement and more adaptive organizational outcomes. Building on Simon’s (1955) foundational notion of bounded rationality, it is well established that leaders, constrained by cognitive limitations, often satisfice rather than pursue optimal decisions, relying on heuristics to simplify complexity (Bazerman & Moore, 2012). Subsequent empirical research has elaborated on this framework, demonstrating how biases such as overconfidence, confirmation bias, anchoring and status quo bias systematically distort leadership decision-making (Powell et al., 2011; Hodgkinson et al., 2023). These distortions are particularly problematic in strategic contexts, where high stakes amplify the need for accuracy. Beshears and Gino (2015) argue that leaders act as “decision architects” by intentionally structuring the contexts in which decisions are made. By designing choice environments that anticipate cognitive biases and bounded rationality, leaders can promote more ethical, effective, and adaptive decision-making processes across their organizations. Kahneman (2011), building on the dual-process framework, provides further insight into leadership cognition by differentiating between the fast, intuitive process 1 and the slow, analytical process 2 modes of thinking. Leaders frequently operate under time pressures that favor the expedient but error-prone process 1 (Bazerman & Tenbrunsel, 2011). A large part of the literature suggests that people engage in very different behaviors when they are acting under time pressure, as compared to more slow-paced decisions, for example, in the social domain (Capraro et al., 2017; Capraro, 2024). Effective leadership therefore entails cultivating the capacity to engage process 2 deliberately, particularly when navigating complex, contentious, or high-impact decisions (Beshears & Gino, 2015). Practical tools such as checklists, feedback loops and decision audits serve to facilitate this cognitive shift, fostering more reflective and rational decision-making processes.

The behavioral economics perspective further enriches this discourse through the concept of choice architecture, as articulated by Thaler and Sunstein (2008). Here, leadership is framed as the intentional structuring of decision environments to “nudge” individuals toward improved outcomes without constraining their freedom. This approach has gained traction within organizational leadership, offering concrete strategies for behavioral influence. For example, leaders may employ opt-out defaults to encourage participation in beneficial programs or strategically time communications to boost engagement (Ebert & Freibichler, 2017).

Houdek and Koblovský’s (2017) reconceptualization of organizational behavior incorporates behavioral economics principles such as reciprocity, intrinsic motivation, and reference-dependent preferences, moving beyond traditional models. This perspective aligns with Ariely’s (2008) distinction between social norms—grounded in trust, relationships, and reciprocity—and market norms—predicated on monetary exchanges and formal contracts. Ariely (2008) emphasizes that conflating these two types of norms can undermine trust and reduce engagement, as the motivational mechanisms underpinning each are fundamentally different. For leaders, discerning when to invoke social versus market norms is critical, as inappropriate blending can undermine trust and engagement. Empirical work by Manthei et al. (2019) supports this assertion, demonstrating through field experiments that the framing of performance feedback significantly influences employee behavior, highlighting the nuanced interplay between motivation and leadership practices.

In shaping decision environments under conditions of uncertainty and bounded rationality, leaders can leverage behavioral influence techniques such as Cialdini’s (2009) six principles of influence—reciprocity, commitment and consistency, social proof, authority, liking and scarcity. These principles enable leaders to guide behavior subtly and effectively. For instance, fostering consistency enhances trust and reinforces organizational commitment, while leveraging social proof capitalizes on group dynamics to facilitate change. Authority and personal likability augment a leader’s persuasive capacity, and invoking scarcity can create a sense of urgency, driving timely action. Together, these strategies underscore the behavioral mechanisms through which leaders can architect organizational culture and decision-making.

This behavioral lens also explains common leadership pitfalls, such as the avoidance of profitable risks driven by loss aversion or the persistence in failing strategies due to escalation of commitment (Kahneman, 2011; Powell et al., 2011). It challenges the classical economic assumption of consistent utility maximization by leaders and followers, advocating instead for more nuanced, behaviorally grounded models of choice under uncertainty. In applying these models, however, organizations need to integrate individual heterogeneity in preferences such as risk and loss aversion.

Empirical evidence supports the efficacy of behavioral interventions in leadership contexts. Nudge-based communication strategies developed by leaders have been shown to enhance participation and collaboration (Smets, 2018). The integration of AI-driven micro-interventions offers real-time feedback that improves leaders’ decision quality and self-regulation (Vecchio & Cavallo, 2019), in line with *hypernudging* and other personalization-based practices (Yeung, 2019; Mills, 2022; Sunstein, 2024). In applied settings such as healthcare and finance, behavioral science interventions like structured and evidence-based strategies designed to change or influence human behavior have demonstrably reduced medical errors, improved patient outcomes and bolstered resilience in crisis leadership scenarios (Liu et al., 2022; Hubbart, 2024).

Nonetheless, important gaps and opportunities for future research remain. Informed estimates suggest that only a small fraction (3%) of leadership research variables are behaviorally measured through objectively observed actions, underscoring the need for more rigorous and comprehensive methodologies (Banks et al., 2023). Emerging theoretical contributions, such as McClanahan’s (2020) dual-strategy theory (hinging upon dominance vs. prestige strategies), warrant extensive empirical validation. Furthermore, the predominance of Western-centric studies calls for broader cross-cultural investigations to ensure the generalizability of behavioral insights across diverse organizational contexts (Enste & Altenhöner, 2021). Addressing these gaps will require longitudinal designs and innovative methods capable of capturing real-time, context-specific leadership behaviors.

## 5. Personalization and Employee Experience

In earlier sections, we have recurrently highlighted the power of personalization to improve the effectiveness of people management practices from the point of view of the organization. Yet, personalization is also an excellent weapon to help the other side of the equation thrive, that is, to enhance the so-called employee experience. As organizations increasingly recognize the strategic importance of employee experience, there is a growing emphasis on moving away from standardized HR practices toward personalized, context-aware interventions. Behavioral economics provides a compelling framework for understanding—and designing—such personalized approaches.

One of the core insights from behavioral science is that individuals differ markedly in their preferences, motivations, and cognitive biases. These differences are not noise to be averaged out, but a signal to be leveraged. For instance, employees vary in their sensitivity to time delays (present bias) or losses (loss aversion), to social comparison (status concerns), and to the perceived meaning of their work (intrinsic motivation). Consequently, two workers exposed to the same policy—be it a feedback system, a reward scheme, or a work-from-home protocol—may experience it in radically different ways.

Behavioral economists have recently started to explicitly argue that one-size-fits-all designs are suboptimal in such settings (Sunstein, 2024; Loewenstein & Chater, 2017). The principle of choice personalization—tailoring default options, communications, or incentives to individual behavioral traits—, however, is not new and has shown promise in improving health, savings, and learning outcomes (Thaler & Sunstein, 2008; Milkman et al., 2011). In the workplace, personalized interventions are now being applied to areas such as onboarding, learning and development, performance feedback, and digital well-being.

Recent experimental research, as synthesized in Espín and Martín (2024) and Espín and de Rus Gutiérrez (2025), supports the feasibility and effectiveness of personalization within organizations. For example, personalized incentive schedules, adapted to individual discount rates and risk preferences, have been shown to increase compliance and engagement in areas such as vaccination (Andreoni et al., 2023). Similarly, Espín et al. (2019) report that aligning incentive timing with employees’ time preferences significantly improved performance in a software development firm—underscoring the motivational value of matching organizational practices to psychological traits. Ultimately, allocating the correct people to the correct place and the right incentives to each one entails that employees face environments where they can thrive and maximize their job satisfaction and well-being.

In fact, personalization plays a central role in shaping the subjective experience of employees. Elements such as perceived autonomy, fairness, recognition and meaningfulness are strong predictors of satisfaction, commitment and retention (Ryan & Deci, 2000; Helliwell & Huang, 2011). Behavioral economics enhances this perspective by emphasizing perception-based mechanisms—such as reference dependence, framing effects and adaptation. For instance, a flexible schedule will be more appreciated if perceived as earned recognition rather than as a generic policy, and a feedback tool will be more motivating if its content and tone align with the employee’s self-concept and confidence level (Gino & Staats, 2016).

Digital technologies have made personalization at scale increasingly feasible. Learning management systems can adapt course content to learner pace and preferences; wellness apps can tailor nudges to behavioral patterns; AI-based recommendation engines can suggest optimal task assignments or break schedules based on cognitive load. Yet this technological personalization must be behaviorally informed to be effective. For example, Ohlwein and Bruno (2025) found that personalized pricing may negatively affect consumers’ perceptions of fairness due to underlying cognitive and emotional processes. As Yeung (2019) warns in her discussion of *hypernudging*, personalization without transparency and consent can undermine trust and autonomy. Behavioral HR systems must therefore balance effectiveness with fairness, agency and explainability.

This notwithstanding, personalization systems often link data to tangible workplace outcomes—such as reward allocation, promotion eligibility, or performance ratings—which can inadvertently create incentives for employees to misrepresent or strategically curate their data. For instance, employees may strategically inflate self-reported engagement scores to avoid undesired interventions. To mitigate this risk, organizations can employ triangulation strategies, combining multiple independent data sources (e.g., behavioral metrics, peer assessments, and performance outcomes) with a particular focus on hard-to-manipulate measures such as those elicited using economic games (Espín, 2025), minimizing the preponderance of self-reported data. Additionally, designing systems where truthful reporting yields long-term benefits—for instance, tailored development opportunities that demonstrably enhance career prospects—can reduce the temptation to “game” the system. Periodic audits and random verification checks might further strengthen data integrity.

An important consideration, highlighted by Espín and de Rus Gutiérrez (2025), is the ethical and strategic advantage of personalization in the public sector and mission-driven organizations. Employees in such contexts often exhibit stronger intrinsic motivations and social preferences, making them particularly sensitive to how policies align with their values. Personalization here is not merely a tool for compliance or efficiency, but a way to respect and engage employees as moral agents and co-constructors of organizational purpose and, ultimately, societal outcomes.

In conclusion, personalization represents a powerful extension of behavioral economics into the domain of employee experience. By integrating diagnostics of individual behavior and preferences into the design of HR practices, organizations can move from managing “resources” to supporting people.

## 6. Organizational Change Initiatives: A Behavioral Perspective

Organizational change initiatives frequently fail to achieve their intended outcomes. Despite significant investments in training, communication, and leadership alignment, the results are often unfinished. An expanding body of behavioral science research suggests that this shortfall may arise from a fundamental misalignment: most change efforts focus excessively on informing or persuading employees, while neglecting the underlying behavioral systems that sustain long-term action.

Behavioral science conceptualizes change not merely as a consequence of knowledge or attitudes but as a process embedded in context, habit formation, and cognitive efficiency. Frameworks such as the COM-B model (Capability, Opportunity, Motivation–Behavior) emphasize that desired behaviors only emerge when individuals possess the necessary skills, operate within supportive environments, and are both consciously and automatically motivated to act (Michie et al., 2011).

Although this perspective has been extensively developed within health interventions (focusing on patients, practitioners, and healthcare systems), it remains underapplied in organizational environments. Early evidence indicates promising potential for transfer across business sectors; however, the existing literature is predominantly conceptual or restricted to health-related contexts, underscoring the need for further empirical validation. The field holds significant promise, but progress requires moving beyond theoretical formulations to design scalable, behaviorally informed interventions applicable to workplace realities.

### 6.1. The Limitations of Traditional Change Models

Conventional approaches to behavioral change in organizations often rely on educational programs, awareness campaigns, or motivational initiatives. While such methods can effectively generate behavioral intentions, they seldom result in enduring behavioral change. As Verplanken and Wood (2022) observe, individuals may endorse behavioral change in principle yet fail to implement it due to entrenched habits or contextual constraints. For instance, training programs frequently show limited transfer to everyday work behavior unless reinforced by environmental supports (Fossey et al., 2019).

A pivotal insight from behavioral science is the well-documented intention–behavior gap. Meta-analytic evidence reveals that substantial changes in intention (Cohen’s d ≈ 0.66) often yield only modest changes in actual behavior (d ≈ 0.36) (Webb & Sheeran, 2006). Thus, “knowing what to do” diverges markedly from “doing it,” particularly within cognitively demanding and distraction-laden organizational contexts.

Prevailing psychological models such as the Theory of Planned Behavior (Ajzen, 1991) and the Health Belief Model (Janz & Becker, 1984), both conceptually transferable to organizational contexts, emphasize rational deliberation and planned decision-making. Nevertheless, these models tend to underrepresent the influence of habits, environmental triggers, and automatic processes (Michie et al., 2011). Consequently, strategies derived from such frameworks often remain conceptually appealing yet practically constrained.

An additional barrier often overlooked is mental effort aversion. Behavioral change demands not only motivation but also significant cognitive resources. Learning new systems, recalling novel procedures, and overriding habitual responses all impose mental effort, something individuals are predisposed to avoid (Otto & Daw, 2019). Recent evidence demonstrates that people systematically avoid cognitive exertion as much as they avoid physical strain or unnecessary expenditure (David et al., 2024). In essence, humans tend to pursue the path of least mental resistance. This aligns with the dual-process theory of decision-making (Kahneman, 2011) and illuminates subtle resistance behaviors such as procrastination, partial compliance, or regression to previous routines. Even when employees support change in principle, the cognitive cost of execution can deter engagement.

From a behavioral design standpoint, the implication is clear: change should be made easier. Processes should be simplified, and new behaviors embedded within existing routines. When the desired behavior becomes the path of least resistance, adoption is substantially more likely.

### 6.2. Habits: The Architecture of Behavior

In organizational settings, much of behavior operates through habit. Employees tend to rely on established routines, “the way things are done here”, which require minimal conscious thought (Wood & Neal, 2007). While this automaticity often enhances efficiency, it simultaneously generates a form of behavioral inertia that resists change.

Two implications arise from this understanding. First, existing habits impede transformation: even when employees intend to behave differently, the familiar cues of their work environment can trigger automatic responses. Under pressure or distraction, individuals typically revert to habitual behaviors (Gardner et al., 2012). Second, new habits must be cultivated to sustain change. Behavioral modification endures only when new practices are repeated within stable contexts until they become automatic. Initial compliance is insufficient; without reinforcement and contextual support, new behaviors rarely persist.

Behavioral reinforcement theory supports this view, emphasizing that sustainable change requires environmental redesign, adding behavioral cues, reducing friction, and aligning rewards, rather than focusing exclusively on internal attitudes or beliefs (Trenz & Keith, 2024).

### 6.3. COM-B: A Behaviorally Informed Model for Organizational Change

The COM-B model provides a structured framework for designing interventions grounded in behavioral science. It posits that behavior (B) emerges from the interaction of Capability (C), Opportunity (O), and Motivation (M) (Michie et al., 2011). Crucially, the model integrates both reflective motivation and automatic processes such as habit formation, thereby advancing beyond frameworks that assume information and persuasion alone suffice. Without the necessary capabilities and a supportive opportunity structure, motivation alone is insufficient to sustain behavioral change.

Applied research illustrates how such behavioral models can inform organizational change. The most effective interventions often modify social and physical opportunities through enabling tools, peer modeling, and environmental restructuring, rather than relying solely on informational campaigns. Much of this evidence originates in the health domain but demonstrates strong potential for cross-sectoral transfer. For example, in healthcare contexts, Fossey et al. (2019) found that staff trained in a new care model reverted to old behaviors unless training was complemented by continuous peer support and environmental cues.

Similarly, Staddon et al. (2016) applied insights from health-related behavioral interventions to identify effective strategies for promoting energy-saving behaviors in office environments. Employing the Behavior Change Wheel (Michie et al., 2014) across 22 studies, they found that interventions emphasizing Enabling (direct support and enhanced control), Environmental Restructuring (automation and modernization of technologies), and Modeling (social influence mechanisms) were the most successful.

In digital contexts, Schnorrenberg et al. (2025) examined sustainability engagement in a banking application that provided users with personalized carbon footprint reports. Using COM-B to analyze user behavior, they found that emotional responses, perceived usefulness, and interface simplicity predicted engagement, with the model explaining over 60% of user variance, highlighting its relevance in complex, technology-mediated environments.

Similarly, McKinlay et al. (2025) identified multiple behavioral determinants, including knowledge, identity, emotions, social networks, and environmental conditions, affecting individuals’ participation in blood pressure checks. Using the Action, Actor, Context, Target, Time (AACTT) framework and the Behavior Change Wheel, they mapped barriers and enablers, reinforcing the utility of COM-B in understanding behavioral systems. Although COM-B has demonstrated considerable success in public health (Koulouvari et al., 2025), its application in organizational contexts remains limited. Preliminary evidence suggests significant promise but also underscores the necessity for more robust empirical testing in workplace settings.

Collectively, these findings illustrate both the potential and the current constraints of behavioral models such as COM-B. While these frameworks offer valuable insights into the mechanisms of change, most studies remain small-scale or context-specific. Advancing this field requires rigorous, longitudinal research and the scaling of behaviorally informed interventions within complex organizational systems. Translating evidence from healthcare into organizational practice represents a promising yet underexplored pathway toward achieving more effective, sustainable change.

## 7. Conclusions and Future Directions

Although this review does not present new empirical results, it offers a structured theoretical consolidation of behavioral economics mechanisms relevant to people management. The integrative framework presented in Figure 1 connects incentive design, decision-making processes, leadership, personalization, and organizational change under a behavioral feedback loop. This system-level perspective clarifies how behavioral parameters can be formally embedded within HR practices and provides a conceptual foundation for future empirical testing.

Behavioral economics offers a powerful and empirically grounded framework to enhance people management by shifting the focus from idealized, rational decision-makers to real individuals—those who are influenced by cognitive biases, social preferences, and contextual cues—, while retaining the formalization of economics research. As highlighted in this review, behavioral insights challenge traditional economic assumptions across key HR domains, from the design of incentives to recruitment, leadership development, and employee experience. Behavioral economics thus represents a unique synthesis: it keeps the explanatory rigor of economic modeling while expanding its scope through the empirical richness of the behavioral sciences. This makes it especially well-suited for advancing research in organizational behavior and the science of people management.

A central insight is that incentives—monetary or not—do not operate in a vacuum; their effectiveness depends on psychological framing, perceptions of fairness, and the interplay with intrinsic motivation. Misaligned or poorly designed incentives can lead to unintended consequences such as entitlement effects or motivational crowding out. Conversely, well-calibrated incentives, sensitive to social norms and individual differences, can foster sustained engagement and performance.

Behavioral economics also exposes the vulnerability of decision-making processes to unconscious bias and heuristics. HR practices such as candidate evaluation, promotions, or terminations can be made more objective and inclusive through behavioral interventions like anonymized assessments, structured criteria, or norm-based nudges. Similarly, leadership is no longer viewed solely through the lens of traits or charisma, but as a behaviorally grounded process of influence and responsiveness to social signals.

Importantly, the rise in digital tools and behavioral data opens new possibilities for personalization at scale. Individual differences in risk tolerance, prosociality, or time preferences—traditionally invisible to managers—can now be measured and incorporated into customized HR interventions, ranging from adaptive onboarding experiences to tailored career development paths. This personalization aligns well with growing expectations for employee-centric and inclusive workplaces.

Implementing personalization, however, requires a considerable volume of individual-level data, and the collection, storage, and analysis of such information entails costs for both employers and employees. These costs are not limited to financial outlays for data infrastructure, analytics platforms, and specialist staff, but also include the time, effort, and cognitive load on employees when completing assessments or providing any type of information. Organizations should weigh these costs against expected benefits through structured cost–benefit analyses and consider phased pilot-scale implementations before investing in large-scale systems.

Lines of work are emerging from across the behavioral sciences that will introduce more effective intervention models in people management. The study of evidence and the usefulness of behaviors and habits to improve interventions in organizational change processes will be a relevant field in the coming years.

Nevertheless, future research should deepen our understanding of how behavioral principles interact with organizational culture, technological change, and broader societal trends. Key questions remain about the ethical boundaries of behavioral interventions at work: How can we ensure transparency and autonomy while leveraging behavioral design? Under what conditions do nudges backfire or lose effectiveness over time? Longitudinal field experiments and interdisciplinary collaborations will be essential to address these challenges.

Ethical concerns regarding data collection and use are also critical. A significant barrier to personalization in people management is employee reluctance to share detailed personal or behavioral data, particularly with their employer. Concerns over confidentiality and potential misuse of information can erode trust, undermining the very engagement that personalization seeks to enhance. To address this, organizations may adopt privacy-by-design principles, ensuring that data collection methods incorporate anonymization techniques when necessary, encryption, and minimal data capture consistent with stated objectives. Informed consent is essential, and communication should specify clearly what data will be collected, why, and how it will be used. In some cases, the involvement of independent third-party data custodians or the deployment of encrypted self-report tools can provide additional assurances and foster greater willingness to participate. A recurrent concern in both research and practice is the “secondary use” of personal data for purposes not originally disclosed at the time of collection. Clear policies and transparent reporting to employees should prevent this concern in advance. In addition, periodic ethics reviews—whether internal or external—can monitor compliance, and adherence to established data protection frameworks, such as the GDPR’s principles of purpose limitation and data minimization, should be non-negotiable.

In synthesizing the previous sections, this review positions behavioral profiling as a unifying mechanism across the domains of incentives, decision-making, leadership, personalization, and organizational change. By systematically identifying individual differences in preferences, motivations, and biases based on behavioral economics modelling, organizations can tailor interventions in each domain with greater precision and fairness. For example, incentive schemes can be aligned with measured loss aversion or time preferences; decision-making frameworks can be adapted to account for social preferences and risk tolerance; leadership styles can be calibrated to follower profiles; personalization can move beyond generic HR practices; and change initiatives can target the behavioral levers most relevant to specific employee segments. In this way, behavioral profiling becomes both a diagnostic and a design tool, enabling a coherent, cross-domain strategy for people management.

While the behavioral economics approach offers substantial promise for improving people management, its application in organizational contexts is not without limitations and risks. Excessive reliance on nudges can lead to unintended consequences such as decision fatigue, manipulation, or the erosion of autonomous choice—sometimes referred to as “dark nudges.” Similarly, hyperpersonalization, if implemented without transparency, may be perceived as unfair or overly intrusive, potentially undermining trust. Behavioral heterogeneity further complicates implementation: interventions that work for one subgroup may be ineffective or even counterproductive for another.

Ethical considerations are central. The collection and use of behavioral data require clear consent protocols and safeguards for privacy. Interventions must be designed with proportionality, transparency, and reversibility in mind. Moreover, there is a need to anticipate potential backfire effects—such as reduced motivation when autonomy is perceived to be constrained—and to evaluate not only short-term behavioral shifts but also long-term cultural impacts. Acknowledging these limitations does not diminish the value of behavioral tools; rather, it ensures their responsible and sustainable integration into organizational practice.

## Data Availability

No new data were created or analyzed in this study.

## Appendix A. Glossary of Concepts

Anchoring Bias—The tendency to rely heavily on the first piece of information offered (the “anchor”) when making decisions.

Behavioral Economics—A field that combines insights from psychology and economics to understand how people actually make decisions, often deviating from purely “rational” models.

Behavioral Profiling—Assessing individuals’ behavioral tendencies (such as risk tolerance or fairness concerns) to guide decisions or tailor interventions.

Bounded Rationality—The idea that people’s decision-making is limited by cognitive constraints, time, and available information.

Choice Architecture—The way in which options are organized and presented to influence decision-making without restricting freedom of choice.

Cognitive Biases—Systematic thinking errors that affect judgment and decision-making through deviations from rationality assumptions, often unconsciously.

COM-B Model—A framework for understanding behavior that emphasizes Capability, Opportunity, and Motivation as necessary components for action.

Crowding-Out Effect—When offering external rewards (like money) reduces a person’s internal motivation to perform a task.

Discount Rate—Standard operationalization of individual time preferences through a parameter that measures the diminishing subjective value of (future) rewards over time.

Escalation of Commitment—Continuing to invest in a failing course of action because of previous investments of time, money, or effort.

Fairness Preferences—Intrinsic valuation of equitable outcomes and fair processes, influencing decision-making even when such choices deviate from purely self-interested economic incentives.

Framing Effect—How the way information is presented (as a gain or a loss, for example) can influence the decisions people make.

Halo Effect—A bias where a positive impression in one area influences overall judgment about a person or thing.

Hypernudging—A dynamic and personalized form of nudging that uses real-time data and AI to continuously adjust interventions.

Implicit Discrimination—Unconscious bias that leads to unfair treatment of individuals based on characteristics like gender or ethnicity.

Inequity Aversion—A dislike of unfair situations, either when receiving less than others or sometimes even when receiving more.

Intention–Behavior Gap—The common difference between what people say they intend to do and what they actually do.

Loss Aversion—The tendency to strongly prefer avoiding losses over acquiring gains of the same size.

Nudges—Subtle interventions in choice architecture that steer people toward certain decisions while keeping all options available.

People Management—The process of recruiting, developing, and managing employees to achieve organizational goals, focusing on performance, engagement, and leadership, among others.

Personalization—Refers to the tailoring of information, choice architecture, or interventions to an individual’s specific characteristics, preferences, and behavioral patterns to enhance decision relevance and effectiveness.

Prospect Theory—A theory explaining how people evaluate potential gains and losses, showing that losses usually feel more significant than equivalent gains.

Reciprocity—The tendency to respond to kind or unkind actions with similar behavior in return.

Relative Performance Incentives—Reward systems based on comparing employees’ results to each other, such as rankings or tournaments.

Risk Preferences—Individuals’ level of tolerance of outcome volatility in decision-making.

Self-Selection—The process by which people choose roles or jobs that match their personal traits, potentially shaping workforce composition.

Social Preferences—Individual tendencies to care about fairness, reciprocity, or the well-being of others in decision-making.

Social Proof—Tendency to copy the actions of others, as they signal the “right” thing to do, especially in ambiguous situations.

Status Quo Bias—A preference for keeping things the same rather than making changes, even when change could be beneficial.

Time preferences—Individual tendencies to value more the short- or the long-term in intertemporal choice.

Transformational Leadership—A leadership style that inspires and motivates followers to exceed expectations by focusing on vision, values, and personal growth.
